# Patterns of Autologous and Nonautologous Interactions between Core Nuclear Egress Complex (NEC) Proteins of α-, β- and γ-Herpesviruses

**DOI:** 10.3390/v12030303

**Published:** 2020-03-11

**Authors:** Sigrun Häge, Eric Sonntag, Eva Maria Borst, Pierre Tannig, Lisa Seyler, Tobias Bäuerle, Susanne M. Bailer, Chung-Pei Lee, Regina Müller, Christina Wangen, Jens Milbradt, Manfred Marschall

**Affiliations:** 1 Institute for Clinical and Molecular Virology, Friedrich-Alexander University of Erlangen-Nürnberg (FAU), 91054 Erlangen, Germany; sigrun.haege@fau.de (S.H.); ericsonntag@web.de (E.S.); pierre.tannig@uk-erlangen.de (P.T.); mueller.regina@uk-erlangen.de (R.M.); christina.wangen@uk-erlangen.de (C.W.); jens.milbradt@lgl.bayern.de (J.M.); 2 Institute of Virology, Hannover Medical School, 30625 Hannover, Germany; borst.eva@mh-hannover.de; 3 Institute of Radiology, University Medical Center Erlangen, 91054 Erlangen, Germany; lisa.seyler@uk-erlangen.de (L.S.); tobias.baeuerle@uk-erlangen.de (T.B.); 4 Fraunhofer Institute for Interfacial Engineering and Biotechnology and Institute for Interfacial Engineering and Plasma Technology IGVP, University of Stuttgart, 70569 Stuttgart, Germany; susanne.bailer@igvp.uni-stuttgart.de; 5 School of Nursing, National Taipei University of Nursing and Health Sciences, 11219 Taipei, Taiwan; chungpei@ntunhs.edu.tw

**Keywords:** α-, β- and γ-herpesviruses, core nuclear egress complexes (NECs), degree of conservation, core NEC interaction properties, autologous vs. nonautologous interactions, multicomponent NEC recruitment of proteins, takeover of activities in vitro and in vivo, functional complementarity

## Abstract

Nuclear egress is a regulated process shared by α-, β- and γ-herpesviruses. The core nuclear egress complex (NEC) is composed of the membrane-anchored protein homologs of human cytomegalovirus (HCMV) pUL50, murine cytomegalovirus (MCMV) pM50, Epstein–Barr virus (EBV) BFRF1 or varicella zoster virus (VZV) Orf24, which interact with the autologous NEC partners pUL53, pM53, BFLF2 or Orf27, respectively. Their recruitment of additional proteins leads to the assembly of a multicomponent NEC, coordinately regulating viral nucleocytoplasmic capsid egress. Here, the functionality of VZV, HCMV, MCMV and EBV core NECs was investigated by coimmunoprecipitation and confocal imaging analyses. Furthermore, a recombinant MCMV, harboring a replacement of ORF M50 by UL50, was analyzed both in vitro and in vivo. In essence, core NEC interactions were strictly limited to autologous NEC pairs and only included one measurable nonautologous interaction between the homologs of HCMV and MCMV. A comparative analysis of MCMV-WT versus MCMV-UL50-infected murine fibroblasts revealed almost identical phenotypes on the levels of protein and genomic replication kinetics. In infected BALB/c mice, virus spread to lung and other organs was found comparable between these viruses, thus stating functional complementarity. In conclusion, our study underlines that herpesviral core NEC proteins are functionally conserved regarding complementarity of core NEC interactions, which were found either virus-specific or restricted within subfamilies.

## 1. Introduction

Herpesviruses are distributed worldwide and are classified into three subfamilies according to their pathogenicity, cell tropism and proliferative characteristics: α-, β- and γ-herpesviruses. All herpesviruses persist after primary infection and can reactivate from this latency. The varicella zoster virus (VZV), a human representative of α-herpesviruses, causes chickenpox and persists in neurons of various ganglions leading to herpes zoster after reactivation. Human cytomegalovirus (HCMV, β-herpesvirus) infection of immunocompetent individuals is mostly asymptomatic and only rarely induces symptoms whereas in immunosuppressed individuals (e.g., transplant recipients or acquired immunodeficiency syndrome (AIDS) patients) infection can cause severe life-threatening symptoms. Importantly, HCMV is the main cause of non-genetic congenital malformations and spontaneous abortions. The most common model in use for studying the principles of β-herpesviral infection and pathogenesis is the murine cytomegalovirus (MCMV). The Epstein–Barr virus (EBV) belongs to the γ-herpesviruses, primarily causes acute infectious mononucleosis, but it is also associated with human cancers such as Burkitt’s lymphoma, nasopharyngeal carcinoma, posttransplant B/T cell lymphomas and gastric cancer. Prevention and therapeutic treatment of herpesviral infections are still a challenging field due to lack of a range of vaccines as well as the adverse side-effects and viral drug resistance frequently induced by approved antiviral drugs.

**Figure 1:**
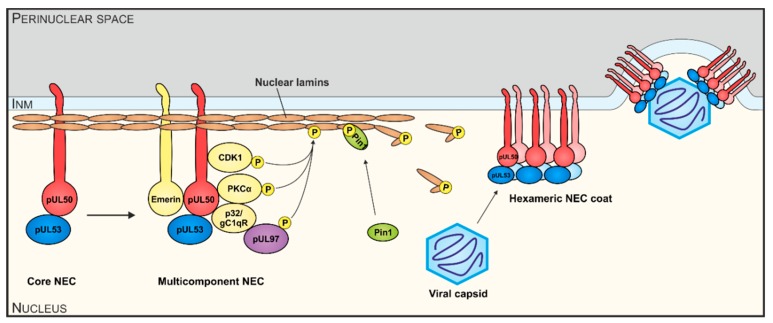
Schematic illustration of functional aspects of human cytomegalovirus (HCMV) nuclear egress complex (NEC) interactions. The HCMV core NEC and multicomponent NEC provide the basis for nuclear lamina as well as membrane-rearranging functions and the formation of a hexameric NEC coat serves as a platform for capsid docking. Viral and cellular protein kinases (pUL97, PKCα, CDK1, others) represent important active components by phosphorylating nuclear lamins A/C, core NEC protein pUL50 and possibly additional NEC constituents.

Herpesviral replication starts in the host cell nucleus, where the viral DNA is amplified and packaged into capsids. Subsequently, capsids are traversing the nuclear envelope (NE), which serves as a physical barrier, into the cytoplasm for further maturation and release. The nucleocytoplasmic transition, termed nuclear egress, is a regulated multistep process independent of the nuclear pore complex and is shared by all herpesviruses. The three main steps are the formation of a multicomponent nuclear egress complex (NEC), the reorganization of the lamina by phosphorylation and the docking and budding of the capsids at the NE (Figure 1). The whole mechanism is balanced by two virus-encoded proteins forming the core NEC. Initially, pUL50 (HCMV) or its homologs in MCMV, EBV and VZV (pM50, BFRF1 or Orf24, respectively), anchored within the inner side of the NE, interacts with the nucleoplasmic cofactor pUL53 (pM53, BFLF2 or Orf27, respectively). The recruitment of several cellular and viral proteins, like protein kinases (pUL97, PKC, CDK1 and possibly others in case of HCMV; [1,2]), by the core NEC leads to the formation of the multicomponent NEC. Site-specific phosphorylation of the nuclear lamins by NEC-associated kinases results in massive rearrangement of the NE and particularly the formation of lamina-depleted areas (LDAs), the sites where viral nuclear capsids gain access to the NE [2,3,4,5]. Additional events of reorganization of the NE, including the formation of a hexameric NEC coat and patch-like lattice within the LDAs apparently serving as a platform for capsid docking, allows the budding of the capsids into the perinuclear space. Hitherto, mainly the regulation of the nuclear egress of individual herpesviruses has been mechanistically investigated and a number of NEC-associated effector proteins have been identified [2,6,7]. Also structural investigations revealed wide-ranging similarities between α- and β-herpesviral NECs [8,9,10,11,12,13,14,15]. The X-ray-based structures of four different α- and β-herpesviral core NECs have been resolved by independent groups [8,12,14,15]. Very recently, we reported the first structure of γ-herpesviral core NEC, namely the 1.75 Å structure of Epstein–Barr virus (EBV) BFRF1-BFLF2, as well as an increased resolution 1.48 Å structure of human cytomegalovirus (HCMV) pUL50-pUL53 [16]. A biochemical comparative analysis of the β- and γ-herpesviral NECs characterized the unique hook-into-groove NEC interaction by applying several different experimental approaches [16]. However, there is still little information, to which extent the functionality of the core NEC, in terms of binding activities, crossviral complementarity and intranuclear transport, is conserved. In this study, we provide novel information on sequence conservation, nuclear rim localization as well as patterns of autologous and nonautologous interactions both in vitro and in vivo, ultimately comparing the functional properties of core NEC proteins of selected members of α-, β- and γ-herpesviruses.

## 2. Materials and Methods 

### 2.1. Cell Culture

Human embryonic kidney epithelial cells (HEK 293T, CRL-3216, ATCC, Manassas, VA, USA), HeLa cells (ATCC) and murine embryonic fibroblasts (own repository of primary cell cultures) were cultivated at 37 °C, 5% CO_2_ and 80% humidity using Dulbecco’s modified Eagle medium (DMEM, 11960044, Thermo Fisher Scientific, Waltham, MA, USA). Cell culture medium was supplemented with 1× GlutaMAX^TM^ (35050038, Thermo Fisher Scientific), 10 μg/mL gentamycin (22185.03, SERVA, Heidelberg, Germany) and 10% fetal bovine serum (FBS, F7524, Sigma Aldrich, St. Louis, MO, USA). 

### 2.2. Plasmids and Transfection 

Transient transfection was performed in 293T cells using polyethylenimine-DNA complexes (Sigma Aldrich) as described previously [17]. HeLa cells were transfected by the use of Lipofectamine 2000 (Thermo Fisher Scientific) according to the manufacturer’s instructions. The following plasmids were used for transfection: pDsRed1-N1 (RFP, Clontech, Kusatsu, Japan), pcDNA-UL50-HA, pcDNA-UL53-Flag [18], pEXPR-IBA5-UL34 (Strep-UL34) and pCR-N-Myc-UL31 [19], pcDNA3.1-HA-BFRF1 and pcDNA3.1-Flag-BFLF2 [20]. Expression plasmids coding for C-terminal HA-tagged or Flag-tagged MCMV, EBV and VZV NEC homologs were generated by standard polymerase chain reaction (PCR) amplification of the respective template DNA produced by infected fibroblasts. MCMV (strain Smith), EBV (strain B95-8) or VZV (strain Oka) were used to generate pcDNA-M50-HA, pcDNA-M53-Flag, pcDNA-BFRF1-HA, pcDNA-BFLF2-Flag, pcDNA-Orf24-HA and pcDNA-Orf27-Flag. Oligonucleotide primers used for PCR were purchased from Biomers (Table S1, Ulm, Germany). After cleavage with the corresponding restriction enzymes, PCR products were inserted into the eukaryotic expression vector pcDNA3.1(+) (Life Technologies, Carlsbad, CA, USA).

### 2.3. Generation of Recombinant Murine Cytomegalovirus (MCMV)

As a template for recombination, we used MCMV Smith strain harboring an insertion of luciferase gene, a deletion of the virus-encoded glycoprotein m157 in order to evade immune response and a repaired *MCK2* gene that is important to promote productive infection of macrophages [21,22,23]. Universal transfer constructs (UTCs) were generated with an insertion of the *Sce*I restriction site, homologous regions required for the recombination step and kanamycin cassette into pUL50 via the unique restriction sites *Bsp*EI (performed by ShineGene Molecular Biotech, Inc., Shanghai, China). Next, UTCs were amplified by PCR using primers 5’-DM50-insUL50 and 3′-DM50-insUL50 (Table S1) and recombination steps were performed (Figure S2) [24]. Recombinant MCMVs were reconstituted by transfection of BACmids into mouse embryonic fibroblasts (MEFs) using FuGENE HD Transfection Reagent (Promega, Madison, WI, USA) according to the manufacturer’s instructions, resulting in the recombinant MCMVs. These were designated as MCMV-WT (parental wild-type M50) and MCMV-UL50 (chimeric exchange M50 vs. UL50). All virus stocks were propagated and titrated on MEFs as described previously [22,25].

### 2.4. Coimmunoprecipitation (CoIP) and In Vitro Assembly-Based CoIP 

For CoIP analysis, 293T cells were seeded into 10 cm dishes with a density of 5 × 10^6^ cells and used for transient transfection with expression plasmids. Two to three days post transfection (d p.t.), CoIP was performed as described previously [26]. Antibody-coupled Dynabeads^TM^ Protein A (10002D, Thermo Fisher Scientific) were used to obtain specific immunoprecipitates and CoIP samples were further analyzed by Western blot (Wb). For CoIPs performed on the basis of protein complexes formed by in vitro assembly, 293T were singly transfected for transient expression using plasmids coding for proteins carrying HA and Flag tags, respectively. One of the two proteins was immunoprecipitated with the tag-specific antibody in an initial IP for 1–2 h. After washing, the sample containing the second tagged protein, or alternatively endogenously expressed untagged protein, was added for assembly and a final CoIP was performed for 1.5–2 h or overnight. 

### 2.5. Indirect Immunofluorescence Assay and Confocal Laser-Scanning Microscopy 

HeLa were grown on coverslips, 2 d p.t. cells were fixed with 4% paraformaldehyde solution (10 min, room temperature) and permeabilized by incubation with 0.2% Triton X-100 solution (15 min, 4 °C). Indirect immunofluorescence staining was performed by incubation with primary antibodies as indicated for 60 min at 37 °C, followed by incubation with dye-conjugated secondary antibodies for 30 min at 37 °C. Cells were mounted with Vectashield Mounting Medium containing DAPI (H-1200, Linaris, Mannheim, Germany) and analyzed using a TCS SP5 confocal laser-scanning microscope (Leica Microsystems, Wetzlar, Germany). Images were processed using the LAS AF software (Leica Microsystems) and Photoshop CS5.

### 2.6. Antibodies

Antibodies used in this study: mAb-HA (Clone 7, H9658, Sigma Aldrich); pAb-HA (Signalway Eurogentec, College Park, MD, USA); mAb-Flag (F1804, Sigma Aldrich); pAb-Flag (F7425, Sigma Aldrich); mAb-Myc (ab9106, Abcam, Cambridge, UK); pAb-Strep (2-1507-001, IBA Lifesciences, Göttingen, Germany); mouse Fc (mouse Fc fragment, 015-000-008, Dianova, Hamburg, Germany); rabbit Fc (rabbit Fc fragment, 011-000-008, Dianova); mAb-Orf27, mAb-Orf24, mAb-UL50.01, mAb-UL97.01 (all kindly provided by Stipan Jonjic and Tihana Lenac Rovis, University of Rijeka, Rijeka, Croatia); mAb-BFRF1 (kindly provided by Alberto Faggioni, Sapienza University of Rome, Rome, Italy); PepAS-M53 (kindly provided by Walter Muranyi, Universitätsklinikum Heidelberg, Heidelberg, Germany); PepAS-M50 (kindly provided by Zsolt Ruzsics, Virology, University of Freiburg, Freiburg, Germany); mAb-β-Actin (A5441, Sigma Aldrich); mAb-mIE1 (Anti-m123/IE1, MCMV, CROMA101, Center for Proteomics, Rijeka, Croatia); pAb-p32 (sc-48795, Santa Cruz Biotechnology, Dallas, TX, USA); anti-mouse Alexa 488 (A-11001, Thermo Fisher Scientific), anti-rabbit Alexa 555 (A-21428, Thermo Fisher Scientific). 

### 2.7. Cytomegalovirus Infection in Cell Culture and Multistep Replication Curve Analysis

Infection experiments were performed at indicated multiplicity of infection (MOI) using parental or recombinant MCMVs. After incubation for 90–120 min at 37 °C, virus inocula were removed and replaced by fresh growth medium. Multistep growth curve analyses of infected MEFs and homogenates of dissected organs were determined by using murine IE1-specific quantitative real-time PCR (qPCR) as described previously [25].

### 2.8. Animal Model

BALB/c mice (6 weeks old) were purchased from Charles River Laboratories (Sulzfeld, Germany), maintained under specific pathogen-free conditions and utilized between 6 and 8 weeks of age. Caging was performed in groups of 5 mice, and body weight was monitored on days 0 and 4 post-infection. Mice were divided into 4 groups with 6 mice each. Two groups were infected with MCMV-WT or MCMV-UL50 and subjected to in vivo imaging at individual days post infection (d p.i.). For infection, animals remained mock-infected or were infected with MCMV at 6.0 × 10^5^ PFU i.p. in a final volume of 100 µL phosphate-buffered saline (PBS). At 2 or 3 d p.i., luciferase signals were quantitated by in vivo imaging. At 4 d p.i., mice were sacrificed. Experimental protocols were reviewed and approved by the Regierung von Mittelfranken, Würzburg, Germany (permit 55.2-2532-2-416; Jun 06, 2017). For in vivo imaging of luciferase-based bioluminescence, mice were anesthetized with isoflurane and placed on a heated bed at 37 °C of an in vivo optical imaging system (IVIS Spectrum, Perkin Elmer, Waltham, MA, USA). The bioluminescence signal in mice was acquired 10 min after intraperitoneal administration of luciferin (150 mg/kg body weight) using an auto-exposure setting with a field of view of 13.2 cm. In the respective regions of interest, the total flux (in photon per second) was acquired.

## 3. Results

### 3.1. Sequence Conservation of Herpesviral Core Nuclear Egress Complex (NEC) Proteins

Recent analysis revealed amazing features of core NEC proteins of α-, β- and γ-herpesviruses, including sequence-specific, structural and functional properties. Functional details of NEC assembly and the regulatory role of NECs in nuclear egress appear closely related and almost congruent between herpesviruses in several aspects, such as recruiting effector proteins responsible for nuclear lamina rearrangement (Figure 1) [2,27]. Similarly, many structural properties of NEC proteins were also found conserved and qualitatively mostly consistent [28,29]. It appeared all the more unexpected that levels of sequence conservation, in terms of amino acid identity, are quite limited or low, even between members within herpesviral subfamilies (Table 1). By comparing ORF-UL50 and ORF-UL53 primary amino acid sequences between herpesviral homologs, we found in both cases a stepwise graduation of levels of conservation. While strains of HCMV showed highly conserved sequences for pUL50 and pUL53 (98.5%–99.5% and 98.4%–100%, respectively), the comparison between HCMVs and primate CMVs (48.0%–54.8% and 56.6%–63.8%), tupaiid herpesvirus 1 (TuHV-1; 39.4%–39.8% and 37.2%–37.4%) or rodent CMVs (30.5%–34.9% and 32.9%–35.6%) showed substantially decreasing conservation levels. Even the comparison with human roseoloviruses (HHV-6A, HHV6-B and HHV-7) underlined the poor NEC amino acid identities with human CMVs (23.3%–25.0% for pUL50 and 29.4%–31.6% for pUL53). This situation strongly suggests that the functional consistency of core NEC proteins is mostly based on common structural features, but is not mirrored by sequence conservation.

### 3.2. Functional Investigation of Herpesviral Core NECs at the Base of Autologous and Nonautologous Interactions

**Figure 2:**
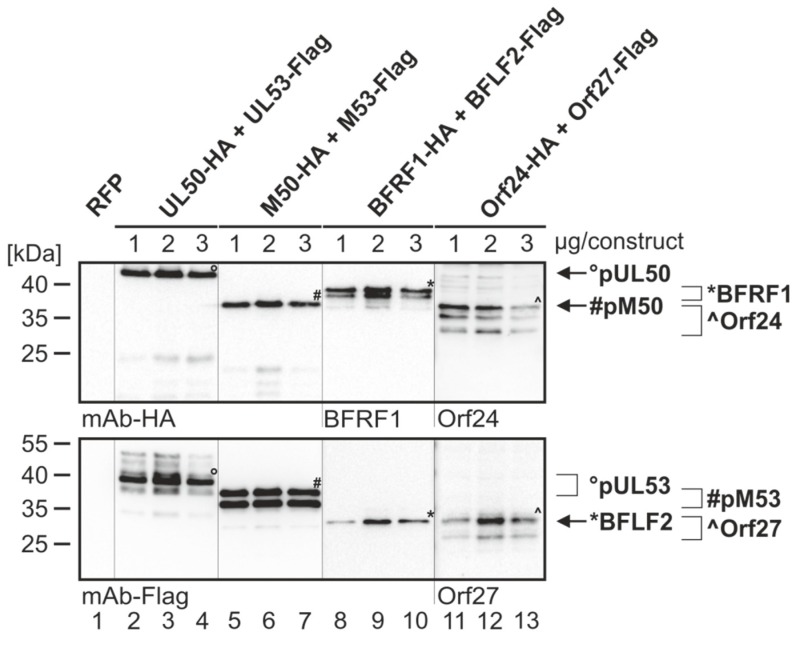
Expression analysis of the core NEC proteins of HCMV, MCMV, EBV and VZV. 293T cells were transiently cotransfected with constructs coding for HA-tagged pUL50, pM50, BFRF1, Orf24 or Flag-tagged pUL53, pM53, BFLF2 and Orf27 with indicated concentrations (1, 2 or 3 µg per construct) in the respective combination, or with pDsRed1-N1 (RFP) as a control. At three d p.t., cells were harvested and lysed. Samples were subjected to standard Wb analysis using tag-specific or protein-specific monoclonal antibodies as indicated. The allocation of protein bands is given by symbols on the right referring to those in the image panels of the figure.

**Figure 3:**
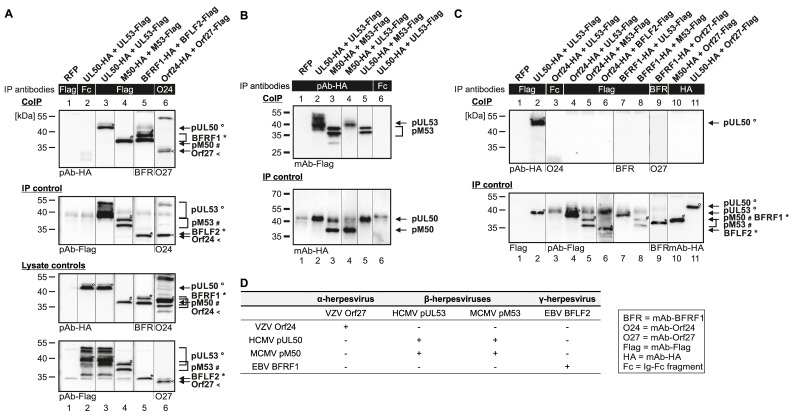
CoIP-based interaction analysis of nonautologous NEC protein pairs derived from HCMV, MCMV, EBV and VZV. 293T cells were transiently transfected with expression plasmids coding for HA-tagged and Flag-tagged versions of NEC proteins as indicated. At three d p.t., cells were lysed and HA- or Flag-tagged proteins were immunoprecipitated using mAb-Flag, mAb-Orf24 (A), pAb-HA (B), mAb-Flag, mAb-BFRF1, mAb-HA (C) or antibody Fc fragment (mouse (A, C) or rabbit (B), as a specificity control). Lysate controls taken prior to the IP and CoIP samples were subjected to standard Wb analysis using tag-specific antibodies as indicated. (**A**) Positive CoIP reactions obtained for autologous pairs of four different herpesviral core NECs. (**B**) Positive CoIP reactions obtained for nonautologous pairs of herpesviral core NECs, when analyzing protein combinations within β-herpesviral subfamily, HCMV and MCMV. (**C**) Negative CoIP reactions obtained for nonautologous pairs of herpesviral core NECs, when analyzing protein combinations between different viral subfamilies. The allocation of protein bands is given by symbols on the right referring to those in the image panels of the figure. (**D**) Combined results obtained from CoIP.

The coding sequences of selected pairs of viral core NEC proteins derived from HCMV (pUL50 and pUL53), MCMV (pM50 and pM53), EBV (BFRF1 and BFLF2) and VZV (Orf24 and Orf27) were cloned into plasmid vectors and used for transient cotransfection experiments in human 293T cells (see protein expression in autologous combinations in Figure 2). The expression pattern demonstrated stable coexpression of all proteins, whereby most of these proteins showed more than one specific band on Western blots (Figure 2, lanes 8–13 upper panels, lanes 2–7 and 11–13 lower panels). This strongly argues for the formation of protein variants, most probably based on posttranslational protein modification. In this regard, data published by our and other research groups demonstrated a pUL53 and pUL50-specific phosphorylation derived from pUL97 and other protein kinase activities [1,13,30]. Interestingly, when comparing the patterns of autologous coexpression to those of single-protein expression or expression in nonautologous, crossviral combinations, a quantitative increase of autologously coexpressed protein pairs was seen in several cases, suggesting a positive effect of core NEC dimerization onto protein stability. This stabilizing effect was visible in terms of both, high signal intensities on Western blots and numbers of positive cells in microscopic investigations, specifically for pUL50–pUL53 and other autologous NEC pairs, as exemplified in additional parallel experiments. As far as the CoIP-based analysis of interaction of analyzed NEC combinations was concerned, a clearly defined result was obtained for all autologous combinations, in that all pUL50 homologs showed strong signals of interaction with their pUL53 homologous counterparts (Figure 3A,B; shown in the two reciprocal settings of CoIP, using either Flag- or HA-specific antibodies for immunoprecipitation). The nonautologous, crossviral combinations, however, did mostly not support interaction with each other, as seen for the combined coexpressions between HCMV, EBV and VZV NEC proteins (Figure 3C). The only exception of nonautologous interaction was the pronounced CoIP of HCMV and MCMV homologs with each other (pUL50/pM50, pUL53/pM53; Figure 3B, lanes 4–5). This indicates that detectable CoIP interaction was restricted to core NEC combinations within the β-herpesviral subfamily (HCMV and MCMV), whereas the analyzed crossviral combinations between different subfamilies were negative (Figure 3D).

### 3.3. Addressing the Question of Crossviral Recruitment of NEC Protein Pairs to the Prominent Rim-Shaped Nuclear Envelope Colocalization 

**Figure 4:**
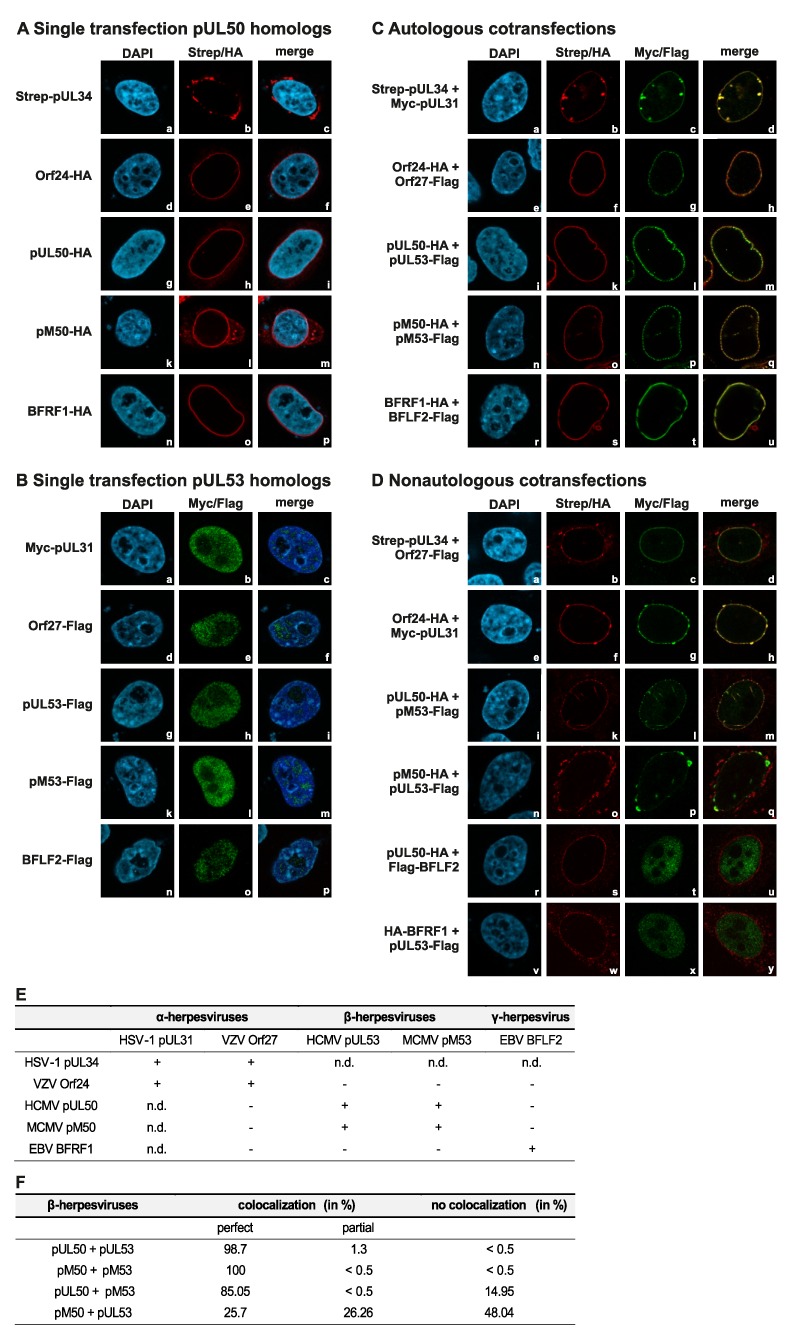
Coexpression of autologous pairs of HSV-1, VZV, HCMV, MCMV and EBV core NEC proteins show perfect nuclear rim colocalization, while nonautologous colocalization is restricted to subfamily-related proteins. HeLa cells were transiently cotransfected with constructs coding for Strep-pUL34, HA-tagged Orf24, pUL50, pM50 and BFRF1 or Myc-pUL31, Flag-tagged Orf27, pUL53, pM53 and BFLF2. Two d p.t., cells were fixed and used for immunostaining with tag-specific antibodies analyzed by confocal imaging. DAPI counterstaining indicated the morphology of nuclei of the respective cells. Single expression of (**A**) pUL50 homologs localized on the nuclear rim and (**B**) pUL53 homologs distributed in the nucleus. (**C**) Colocalization of coexpressed autologous NEC protein pairs on the nuclear rim. (**D**) Coexpressed, colocalizing nonautologous NEC protein pairs of subfamily-related proteins. (**E**) Combined results of autologous and nonautologous combinations. n.d., not determined. (**F**) Quantitation of autologous and nonautologous colocalization of HCMV and MCMV nuclear egress proteins presented as percentages.

Next, we addressed the question whether combinations of coexpressed α-, β- and γ-herpesviral core NEC proteins were able to recruit each other to a nuclear rim colocalization. Using immunofluorescence-based confocal imaging, a clear-cut result was obtained for all singly expressed proteins (Figure 4A,B) and for autologous combinations (Figure 4C). The pUL53 homologs (showing all-over nuclear distribution when singly expressed, Figure 4B) were effectively recruited to a marked nuclear rim colocalization by their pUL50 homolog counterparts (Figure 4C, see merge in right panels d, h, m, q and u; compare to nuclear membrane-anchored rim localization of the singly expressed pUL50 homologs in Figure 4A, central panels). In the case of nonautologous, crossviral combinations, differential patterns were obtained in that pUL50 and pUL53 of HCMV showed perfect nuclear rim colocalization with the MCMV homologs pM50 and pM53, respectively (Figure 4D, m and q). Furthermore, a colocalization of HSV-1 proteins pUL34 and pUL31 with VZV Orf24 and Orf27, respectively, was detectable (Figure 4D, d and h). In contrast, nonautologous combinations between homologs of the different herpesviral subfamilies did not develop colocalization (Figure 4D, u and y; Figure 4E) [16]. The nonautologous colocalization between HCMV and MCMV nuclear egress proteins was additionally quantitated. The autologous combinations showed a colocalization in almost all cells, the nonautologous combination of pUL50 and pM53 colocalized in 85% of the cells. Also, at some lower frequency, the combination of pM50 with pUL53 developed rim colocalization in 51.96% of the cells (perfect colocalization 25.7% or partial 26.26%). These data further underline on the basis of two examples, that crossviral NEC interactions are detectable within herpesviral subfamilies, but not in nonautologous combinations between α-, β- or γ-herpesviral homologs. 

### 3.4. Detectable Binding of Additional, NEC-Associated Proteins to the Analyzed Herpesviral Core NECs

Concerning the four analyzed viral core NECs, their interaction with the cellular NEC-associated multi-ligand binding protein p32/gC1qR and the viral protein kinase pUL97 was analyzed. To this end, an in vitro assembly-based CoIP protocol was applied under conditions established recently [31]. Endogenous levels of p32/gC1qR (p32) and transiently expressed pUL97-Flag were used for in vitro assembly with cellular lysates containing the transiently coexpressed core NECs as indicated (Figure S1). Notably, the p32/gC1qR association of MCMV- and HCMV-specific NECs correlated with a positive signal of the in vitro assembly of transiently expressed pUL97 kinase (Figure S1). The finding was consistent with the previously described p32/gC1qR-bridging function between pUL97/pM97 and these two types of NECs [32,33,34,35]. Further assembly-based CoIPs revealed a surprisingly strong property of pM53 to associate with human p32/gC1qR. This suggested the formation of higher-order complexes with related NEC proteins, such as pUL53. 

**Figure 5:**
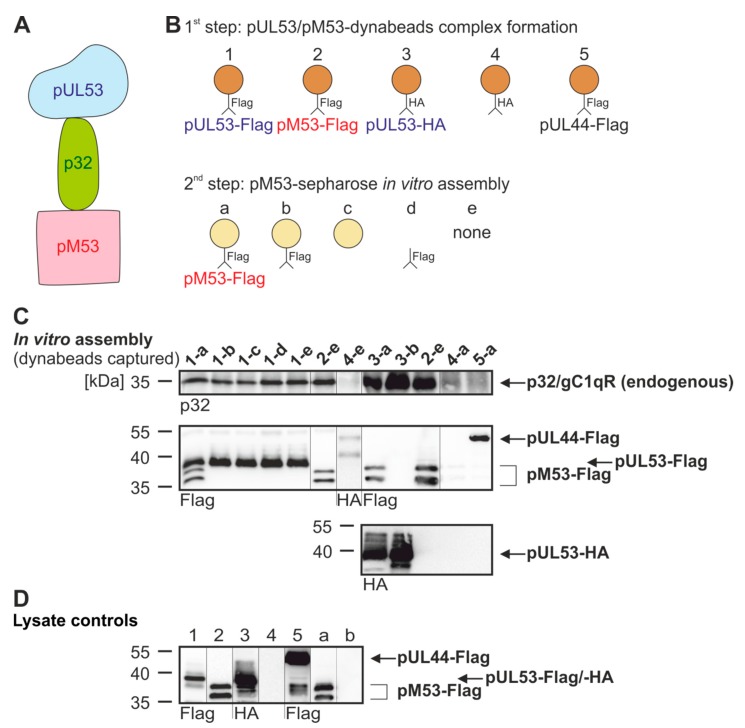
Assembly-based CoIP for interaction of pUL53, pM53 and p32/gC1qR. (**A**) Complex formation of pUL53 and pM53 bridged by p32/gC1qR. (**B**) Schematic overview of the performed experiment. In a first step, pUL53 or pM53 (or pUL44 as a negative control) formed complexes with p32/gC1qR and was immunoprecipitated using dynabeads. In a second step, pM53 was immunoprecipitated with sepharose beads. (**C**) 293T cells were transiently transfected with HA-tagged pUL53, Flag-tagged pUL53, pM53 or pUL44. At two d p.t., cells were lysed and Flag-tagged pUL53, pM53, pUL44 or HA-tagged pUL53 was immunoprecipitated using mAb-Flag or -HA/dynabeads (orange), whereas pM53 was also immunoprecipitated using mAb-Flag/sepharose beads (yellow). Subsequently, settings 1–5 were incubated under the following conditions of CoIP overnight: (a) pM53-Flag immunoprecipitated by sepharose beads, (b) mAb-Flag linked to sepharose beads, (c) sepharose beads alone, (d) mAb-Flag alone or (e) none of these. Thereafter, dynabeads were separated and subjected to standard Wb analysis using tag- or protein-specific antibodies as indicated. (**D**) Lysate controls were taken prior to the IP, analyzed and immunostained by SDS-PAGE/Wb as indicated.

The scenario was further addressed by analyzing the dynamic association of p32/gC1qR with both, HCMV pUL53 and MCMV pM53, in a two-step in vitro assembly procedure (Figure 5A,B). As a 1^st^ step, pUL53-Flag or -HA was expressed by transient transfection and the postulated intracellular complexes formed with endogenous p32/gC1qR were harvested by CoIP with Flag- or HA-specific antibody/dynabeads (orange, Figure 5B, 1–5). In a 2^nd^ step, separately expressed and immunoprecipitated pM53-Flag/sepharose beads (yellow) were added and coincubated (Figure 5C, lane 1-a). In independent reactions, pUL53-Flag/dynabeads were coincubated with further reagents, i.e., mAb-Flag-linked sepharose beads (lane 1-b), sepharose beads alone (lane 1-c), mAb-Flag alone (lane 1-d) or none of these (lane 1-e). In lanes 2-e (in duplicate), an additional control with pM53-Flag expression alone was included, in the absence of any assembly or competition agent. Further settings were a complete negative control (lane 4-e), tag-specificity controls (lanes 3-a, 3-b and 4-a) and a viral protein negative control (lane 5-a). This procedure was performed to address the question whether pM53 interferes in a competitive manner with pUL53-p32/gC1qR interaction or whether pM53 and pUL53 are able to associate with each other. Association could be based on heterodimerization or p32/gC1qR-bridged higher-order complexes (Figure 5A). Notably, for p32/gC1qR a pronounced tendency to form dimeric, trimeric, hexameric and further complex assemblies, dependent on environmental conditions, has been described [36]. On this basis, the experimental data shown in Figure 5 illustrated the complex properties of protein interaction exerted by pUL53, pM53 and p32/gC1qR. This assembly-based CoIP setting detected the non-competitive, additive binding of p32/gC1qR and pM53-Flag when pUL53-Flag or -HA was immunoprecipitated by the use of mAb-Flag/dynabeads. No competitive effect on the interaction pUL53-p32/gC1qR was detectable, neither through pM53 nor through any of the control coincubations. Thus, the result strongly suggested a complex formed by pUL53-p32/gC1qR-pM53. However, on this stage, no distinction could be made between a putative potency of pUL53-pM53 heterodimerization or the in vitro formation of a p32/gC1qR-bridged complex.

### 3.5. Chimeric Murine Cytomegalovirus (MCMV) UL50 Attains Wild-Type Levels of Viral Protein Production and Replication in Cultured Cells

**Figure 6:**
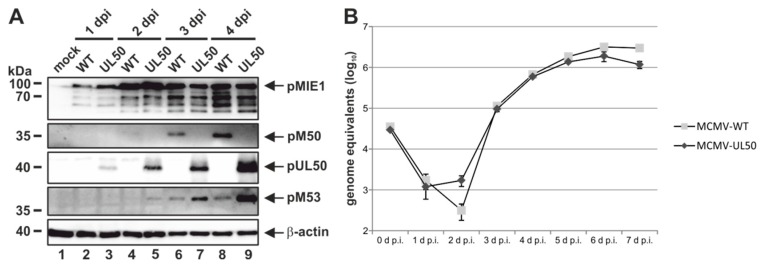
HCMV pUL50 mediates efficient viral protein expression and replication in chimeric MCMV. MEFs were infected with wild-type MCMW-WT and recombinant MCMV-UL50 at an (A) MOI of 1 or (B) MOI of 0.01. (**A**) At various time points, cells were lysed and protein expression was analyzed using protein-specific antibodies. (**B**) Viral supernatants were harvested at the indicated time points and viral genome equivalents released into the supernatant were determined by murine IE1-specific quantitative real-time PCR (qPCR). Each infection was performed in triplicate and mean values and standard deviations are shown.

In order to address the question whether HCMV core NEC protein pUL50 can functionally replace MCMV pM50 in the context of replication in cultured cells or the natural animal host, chimeric MCMV were generated by a red recombination system [24,37]. As a template for recombination, a recombinant of the MCMV Smith strain was used, harboring a luciferase gene insertion, a deletion of the virus-encoded immunomodulatory glycoprotein m157 as well as a repaired *MCK2* gene important for infection of macrophages [21,22,23]. Recombination steps used for the generation of MCMV-UL50 are illustrated in Figure S2. In order to keep the coding capacity of ORF *M49*, the majority of ORF *M50* was replaced by an UTC that carried the ORF *UL50*, the positive selection marker and regions important for homologous recombination. The virus was reconstituted from the BACmids and propagated on MEFs. To investigate the functional importance of pUL50, MEFs were infected with MCMV-WT or chimeric MCMV-UL50 to perform Wb kinetic experiments (Figure 6A). At consecutive time points, murine IE1 protein (pMIE1) production was detectable at equal quantities for the two viruses starting from 1 d p.i. (Figure 6A, upper panel). Notably, the expression of pUL50 was observed at 1 d p.i. (MCMV-UL50), whereas pM50 expression (MCMV-WT) was first detected at 2 d p.i. (Figure 6A, middle panels). Furthermore, as an interesting finding, pM53 expression started at 2 d p.i. in MCMV-UL50-infected cells (even slightly earlier than MCMV-WT), and additionally, pM53 expression was markedly increased in the presence of pUL50 (Figure 6A, panel pM53, lanes 5, 7 and 9) compared to parental viral pM50 (lanes 4, 6 and 8). Next, the replication kinetics of chimeric MCMV-UL50 was analyzed by a multistep replication curve (Figure 6B; MEFs infected with MCMVs at MOI 0.01). Aliquots of cell supernatants were collected at various time points after infection and subjected to qPCR to determine MIE1-specific genome equivalents. Strikingly, MCMV-UL50 exhibited mostly wild-type-like characteristics of genome production and release, only showing slight decrease in quantitative terms, indicating that viral replication was not impaired by the M50-UL50 genetic exchange. Combined, these data illustrate that pUL50 is able to replace pM50 in vitro by fully supporting MCMV protein, genome and virus production.

### 3.6. Experimental Infection of Mice with Chimeric MCMV-UL50 Confirms the Functional Conservation of MCMV/Human Cytomegalovirus (HCMV) Core NEC Proteins in Vivo

**Figure 7:**
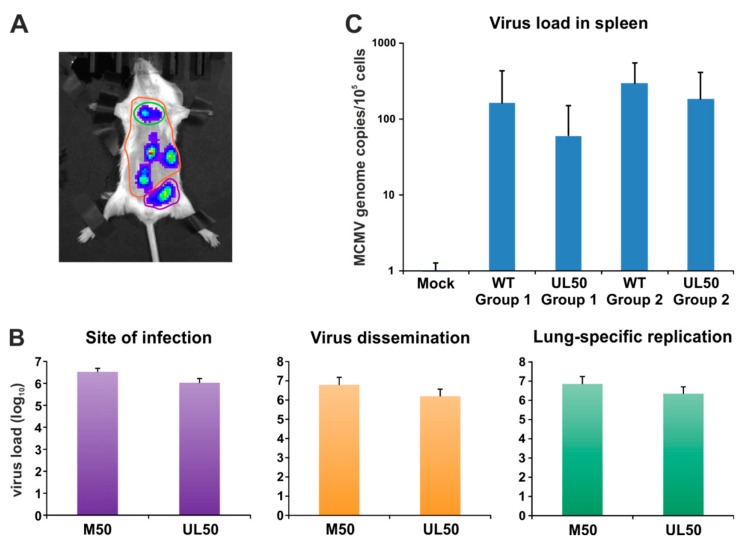
Quantitation of primary and secondary replication. (**A**) Specific regions of interest were selected for site of infection (purple), virus dissemination (orange) and lung-specific replication (green). (**B**) Evaluation was performed using Living Image 4.5. (**C**) Viral genome equivalents in the spleen were determined by murine IE1-specific quantitative real-time PCR.

Finally, we addressed the question whether nonautologous expression of pUL50 by chimeric MCMV-UL50 in mice was capable of promoting normal levels of viral in vivo replication and organ dissemination. To this end, groups of six BALB/c mice each were infected with chimeric MCMVs (two mice remained uninfected, mock control). Firstly, the sites of in vivo replication and virus dissemination were analyzed 2 d and 3 d p.i (Figure S3). For this purpose, 150 mg/kg body weight luciferin was injected intraperitoneally (i.p.) and bioluminescence signals were measured after 10 min. At 2 d p.i., viral replication was detectable at the sites of infection in all groups (Figure S3A); virus spread to the lung was reserved to 33% of MCMV-WT- and 50% of MCMV-UL50-infected mice. At 3 d p.i., all mice infected with MCMV-WT exhibited lung-specific replication (100%); virus spread to various organs (e.g., spleen and liver) within the abdominal area occurred in 84% (Figure S3B). In comparison, 84% of mice infected with MCMV-UL50 displayed lung-specific replication and virus dissemination was detectable in 66%. In conclusion, pUL50 expressed by chimeric MCMV was able to promote virus replication and dissemination in mice, albeit quantitatively reduced to some extent, but with almost MCMV-WT-like characteristics. In addition, we determined parameters of body weight and mouse behaviour. The average initial weight (day 0) was 18.5 g for each group. All MCMV-infected animals presented reduced physical activity and slightly ruffled fur as a symptomatic sign of active viral replication. In order to quantitate MCMV-specific bioluminescence signals, we focused on three distinct areas of the murine torso, namely the site of infection (primary replication), viral organ dissemination (secondary replication) and lung-specific replication (Figure 7A). Intriguingly, all quantitated areas revealed an almost wild-type-like viral load in MCMV-UL50-infected mice (Figure 7B). Furthermore, the analysis of viral load was extended to other organs and methods. Spleen tissues were quantitated by qPCR showing very similar quantities of viral load for both viruses, comparing MCMV-WT with MCMV-UL50 infected mice (Figure 7C). Thus, the chimeric substitution of pM50 by pUL50 efficiently allowed for systemic infection in this animal model including the dissemination to permissive organs.

## 4. Discussion

This study provides novel insights into the interaction properties of VZV, HCMV, MCMV and EBV core NECs, in particular into their biochemical properties of core NEC binding in vitro and in vivo, and their intranuclear rim recruitment and association with additional regularly factors. Specific focus was put on the capacity of functional complementation between HCMV and MCMV core NEC proteins, also including recombinant viruses investigated in infected mice. Combined, data indicate the following: (i) primary sequences of core NEC proteins are poorly conserved when comparing the range spanning over α-, β- and γ-herpesviruses, (ii) crossviral, nonautologous NEC interactions were only identified for closely related NEC pairs (such as those within α- and β-herpesviral subfamilies, like the analyzed examples of NEC proteins of HSV-1 and VZV or HCMV and MCMV, respectively), (iii) the NEC binding properties, intranuclear rim recruitment and patterns of in vitro assembly with NEC-associated proteins further suggest a virus specificity, (iv) the property to form multicomponent NECs is a general characteristic among herpesviruses, and (v) recombinant MCMV-UL50 demonstrated functional complementarity of pUL50 and pM50 in vitro and in vivo.

Previous reports provided important information on the functionality, composition and structure of α-, β- and γ-herpesviral NECs [1,2,6,7,14,38,39]. Initial data were provided by Leigh et al. [11] that a nonautologous interaction is principally detectable between HCMV pUL53 and MCMV pM50, using a synthetic N-terminal peptide of pUL53 in its potential to bind to a truncated version of pM50 recombinantly expressed in *E. coli* as measured by NMR analysis. In the present study, this initial finding was expanded by using full-length proteins transiently expressed in human cells as measured by nonautologous CoIP, confocal colocalization and an in vivo assessment of functional replacement. Concerning NEC functional aspects, previous studies on a number of different herpesviruses consistently characterized the two core proteins of NEC as a heterodimer basically essential for viral replication. Since in all cases analyzed, one of the two NEC proteins resides in the inner nuclear membrane (INM) as a type II, tail-anchored membrane protein (e.g., HCMV pUL50), in a position capable to recruit its heterodimeric partner carrying a classical bipartite nuclear localization signal (NLS) to a prominent NEC nuclear rim localization. This conformity of the NEC opened the theoretical possibility that such corecruitment might even occur between nonautologous NEC pairs, for instance upon a coinfection of individual cells with two different herpesviruses. However, data of the present study illustrated that crossviral, nonautologous NEC interaction is very unlikely, at least this was not supported by evidence through our coexpression- or in vitro assembly-based model systems. On a structural basis, the mode of high-affinity core NEC interaction could be explained by the fact that NEC protein pairs adopt a unique type of binding structure elements responsible for a hook-into-groove interaction. These elements were identified for the first time in the x-ray-based study of the HCMV core NEC, leading to the identification of a hook-like N-terminal extension of pUL53 and a helical groove as a part of the globular domain of pUL50 [14]. Parallel investigations on additional α- and β-herpesviral NECs confirmed this finding [8,11,12,15]. Due to the situation that these elements were structurally highly similar and basically conserved in their 3D shapes, a fact which appeared amazing on the basis of poorly conserved primary sequences, a nonautologous mode of hook-into-groove interaction appeared possible. Our initial data derived from coexpression experiments with domain swap constructs, in which distinct parts of the hook element were artificially exchanged between HCMV and EBV (pUL53::BFLF2 fusions), indicated that very little variability in the hook element is acceptable for retaining a detectable level of high-affinity interaction with the respective groove proteins. In most cases of domain swap, the autologous hook element lost reactivity in NEC interaction [16]. A third aspect considered as potentially facilitating nonautologous NEC interaction was seen in the recruitment of a number of NEC-associated host proteins, a property known for all herpesviral NECs analyzed so far. Interestingly, the composition of these multicomponent NECs is only partially consistent between the individual herpesviruses, and is not even identical between members of the same subfamily [2]. In the case of mass spectrometry-based proteomics analyses performed on HCMV and MCMV, both identical and nonidentical components of their multicomponent NECs have been identified [32,35]. One interesting and consistent finding was the presence of the cellular multi-ligand binding protein p32/gC1qR in α-, β- and γ-herpesviral NECs [33,35,40,41]. Our data provided additional evidence that p32/gC1qR is able to interact with HCMV- and MCMV-specific core NEC proteins (Figure 5 and Figure S1). In this context, it has to be stressed that our current data indicate that HCMV ORF UL50 can be transferred in a functionally intact manner into the MCMV genetic background. Hereby, a similarity in the functional importance of the HCMV and MCMV core NEC proteins pUL50 and pM50 was clearly stated, both in the murine-cultured cell system as well as in infected animals. Thus, this part provides evidence for the in vivo functional complementation of pM50 by pUL50 by using chimeric viruses. This setting indicated that no impairment in protein expression, replication efficiency and organ dissemination was detectable when compared to the parental virus. Beyond the scope of the study, this chimeric CMV model might serve as a tool to further investigate molecular aspects of NEC–host interaction including pUL50 mutants, protein-protein interactions and the study of NEC-inhibitory small molecules. The combined conclusions drawn from findings presented here and in previous reports illustrate that herpesviral core NEC proteins are functionally conserved and it is strongly suggestive that complementarity of core NEC interactions are either virus-specific or restricted within subfamilies. As a future perspective, our study provides a refined functional model that is applicable as the basis for core NEC investigations, comparisons between NEC functions and studies investigating the potency of targeting antiviral drugs towards herpesviral core NEC proteins.

## Figures and Tables

**Table 1:**
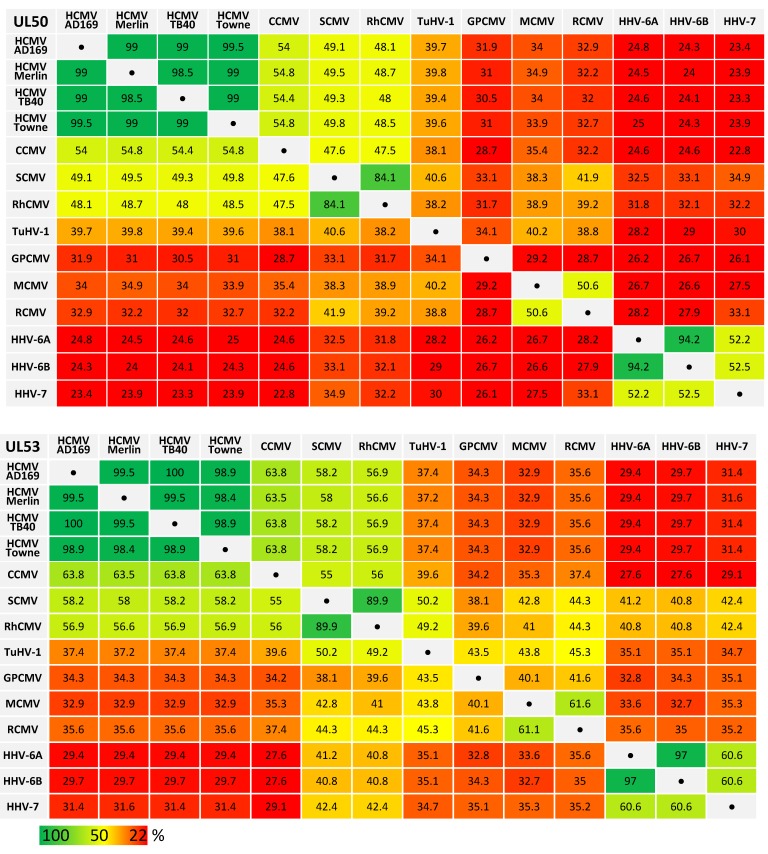
Amino acid sequence identities (%) of human and animal β-herpesviral pUL50 and pUL53 homologs

**Sequences of pUL50 homologs:** HCMV AD169, human cytomegalovirus, strain AD169, P16791; HCMV Merlin, human cytomegalovirus, strain Merlin, Q6SW81; HCMV TB40, human cytomegalovirus, strain TB40, A8T7C7; HCMV Towne, human cytomegalovirus, strain Towne, B9VXL9; CCMV, Chimpanzee cytomegalovirus, Q8QS38; RhCMV, Rhesus cytomegalovirus, Q2FAN6; SCMV, Simian cytomegalovirus, strain Colburn, G8XTV6; TuHV-1, Tupaiid herpesvirus 1, strain 1, NP_116404; GPCMV, Guinea pig cytomegalovirus, strain CIDMTR, U6H6P9; MCMV, Murine cytomegalovirus, strain Smith, D3XDN8; RCMV, Rat cytomegalovirus, strain Maastricht, Q9DWE0; HHV-6A, Human herpesvirus 6A, strain Uganda-1102, P52465; HHV-6B, Human herpesvirus 6B, strain Z29, Q9QJ35; HHV-7, Human herpesvirus 7, strain JI, P52466. **Sequences of pUL53 homologs:** HCMV AD169, human cytomegalovirus, strain AD169, P16794; HCMV Merlin, human cytomegalovirus, strain Merlin, F5HFZ4; HCMV TB40, human cytomegalovirus, strain TB40, A8T7D2; HCMV Towne, human cytomegalovirus, strain Towne, B9VXM2; CCMV, Chimpanzee cytomegalovirus, Q8QS35; RhCMV, Rhesus cytomegalovirus, O71122; SCMV, Simian cytomegalovirus, strain Colburn, G8XTV9; TuHV-1, Tupaiid herpesvirus 1, strain 1, Q91TN5; GPCMV, Guinea pig cytomegalovirus, strain CIDMTR, U6H9V2; MCMV, Murine cytomegalovirus, strain Smith, D3XDP1; RCMV, Rat cytomegalovirus, strain Maastricht, Q9DWD7; HHV-6A, Human herpesvirus 6A, strain Uganda-1102, P28865; HHV-6B, Human herpesvirus 6B, strain Z29, Q9WT27; HHV-7, Human herpesvirus 7, strain JI, P52361.

## Supplementary Materials

The following are available online at https://www.mdpi.com/1999-4915/12/3/303/s1: Table S1. Oligonucleotide primers used in this study, Figure S1: Assembly-based CoIP for core NECs with p32/gC1qR and pUL97, Figure S2: Schematic representation of the generation of recombinant MCMV, Figure S3: In vivo detection of virus replication.

## References

[1] Sonntag E., Milbradt J., Svrlanska A., Strojan H., Hage S., Kraut A., Hesse A.M., Amin B., Sonnewald U., Coute Y. (2017). Protein kinases responsible for the phosphorylation of the nuclear egress core complex of human cytomegalovirus. J. Gen. Virol..

[2] Marschall M., Muller Y.A., Diewald B., Sticht H., Milbradt J. (2017). The human cytomegalovirus nuclear egress complex unites multiple functions: Recruitment of effectors, nuclear envelope rearrangement, and docking to nuclear capsids. Rev. Med. Virol..

[3] Milbradt J., Webel R., Auerochs S., Sticht H., Marschall M. (2010). Novel mode of phosphorylation-triggered reorganization of the nuclear lamina during nuclear egress of human cytomegalovirus. J. Biol. Chem..

[4] Milbradt J., Hutterer C., Bahsi H., Wagner S., Sonntag E., Horn A.H., Kaufer B.B., Mori Y., Sticht H., Fossen T. (2016). The Prolyl Isomerase Pin1 Promotes the Herpesvirus-Induced Phosphorylation-Dependent Disassembly of the Nuclear Lamina Required for Nucleocytoplasmic Egress. PLoS Pathog..

[5] Milbradt J., Sonntag E., Wagner S., Strojan H., Wangen C., Lenac Rovis T., Lisnic B., Jonjic S., Sticht H., Britt W.J. (2018). Human Cytomegalovirus Nuclear Capsids Associate with the Core Nuclear Egress Complex and the Viral Protein Kinase pUL97. Viruses.

[6] Roller R.J., Baines J.D. (2017). Herpesvirus Nuclear Egress. Adv. Anat. Embryol. Cell Biol..

[7] Bailer S.M. (2017). Venture from the Interior-Herpesvirus pUL31 Escorts Capsids from Nucleoplasmic Replication Compartments to Sites of Primary Envelopment at the Inner Nuclear Membrane. Cells.

[8] Bigalke J.M., Heldwein E.E. (2015). Structural basis of membrane budding by the nuclear egress complex of herpesviruses. EMBO J..

[9] Bigalke J.M., Heuser T., Nicastro D., Heldwein E.E. (2014). Membrane deformation and scission by the HSV-1 nuclear egress complex. Nat. Commun..

[10] Hagen C., Dent K.C., Zeev-Ben-Mordehai T., Grange M., Bosse J.B., Whittle C., Klupp B.G., Siebert C.A., Vasishtan D., Bauerlein F.J. (2015). Structural Basis of Vesicle Formation at the Inner Nuclear Membrane. Cell.

[11] Leigh K.E., Sharma M., Mansueto M.S., Boeszoermenyi A., Filman D.J., Hogle J.M., Wagner G., Coen D.M., Arthanari H. (2015). Structure of a herpesvirus nuclear egress complex subunit reveals an interaction groove that is essential for viral replication. Proc. Natl. Acad. Sci. USA.

[12] Lye M.F., Sharma M., El Omari K., Filman D.J., Schuermann J.P., Hogle J.M., Coen D.M. (2015). Unexpected features and mechanism of heterodimer formation of a herpesvirus nuclear egress complex. EMBO J..

[13] Milbradt J., Auerochs S., Sevvana M., Muller Y.A., Sticht H., Marschall M. (2012). Specific residues of a conserved domain in the N terminus of the human cytomegalovirus pUL50 protein determine its intranuclear interaction with pUL53. J. Biol. Chem..

[14] Walzer S.A., Egerer-Sieber C., Sticht H., Sevvana M., Hohl K., Milbradt J., Muller Y.A., Marschall M. (2015). Crystal Structure of the Human Cytomegalovirus pUL50-pUL53 Core Nuclear Egress Complex Provides Insight into a Unique Assembly Scaffold for Virus-Host Protein Interactions. J. Biol. Chem..

[15] Zeev-Ben-Mordehai T., Weberruss M., Lorenz M., Cheleski J., Hellberg T., Whittle C., El Omari K., Vasishtan D., Dent K.C., Harlos K. (2015). Crystal Structure of the Herpesvirus Nuclear Egress Complex Provides Insights into Inner Nuclear Membrane Remodeling. Cell Rep..

[16] Muller Y.A., Hage S., Alkhashrom S., Hollriegl T., Weigert S., Dolles S., Hof K., Walzer S.A., Egerer-Sieber C., Conrad M. (2020). High-resolution crystal structures of two prototypical beta- and gamma-herpesviral nuclear egress complexes unravel the determinants of subfamily specificity. J. Biol. Chem..

[17] Schregel V., Auerochs S., Jochmann R., Maurer K., Stamminger T., Marschall M. (2007). Mapping of a self-interaction domain of the cytomegalovirus protein kinase pUL97. J. Gen. Virol..

[18] Milbradt J., Auerochs S., Marschall M. (2007). Cytomegaloviral proteins pUL50 and pUL53 are associated with the nuclear lamina and interact with cellular protein kinase C. J. Gen. Virol..

[19] Ott M., Tascher G., Hassdenteufel S., Zimmermann R., Haas J., Bailer S.M. (2011). Functional characterization of the essential tail anchor of the herpes simplex virus type 1 nuclear egress protein pUL34. J. Gen. Virol..

[20] Lee C.P., Liu P.T., Kung H.N., Su M.T., Chua H.H., Chang Y.H., Chang C.W., Tsai C.H., Liu F.T., Chen M.R. (2012). The ESCRT machinery is recruited by the viral BFRF1 protein to the nucleus-associated membrane for the maturation of Epstein-Barr Virus. PLoS Pathog..

[21] Jordan S., Krause J., Prager A., Mitrovic M., Jonjic S., Koszinowski U.H., Adler B. (2011). Virus progeny of murine cytomegalovirus bacterial artificial chromosome pSM3fr show reduced growth in salivary Glands due to a fixed mutation of MCK-2. J. Virol..

[22] Klenovsek K., Weisel F., Schneider A., Appelt U., Jonjic S., Messerle M., Bradel-Tretheway B., Winkler T.H., Mach M. (2007). Protection from CMV infection in immunodeficient hosts by adoptive transfer of memory B cells. Blood.

[23] Wagner F.M., Brizic I., Prager A., Trsan T., Arapovic M., Lemmermann N.A., Podlech J., Reddehase M.J., Lemnitzer F., Bosse J.B. (2013). The viral chemokine MCK-2 of murine cytomegalovirus promotes infection as part of a gH/gL/MCK-2 complex. PLoS Pathog..

[24] Tischer B.K., von Einem J., Kaufer B., Osterrieder N. (2006). Two-step red-mediated recombination for versatile high-efficiency markerless DNA manipulation in Escherichia coli. Biotechniques.

[25] Lorz K., Hofmann H., Berndt A., Tavalai N., Mueller R., Schlotzer-Schrehardt U., Stamminger T. (2006). Deletion of open reading frame UL26 from the human cytomegalovirus genome results in reduced viral growth, which involves impaired stability of viral particles. J. Virol..

[26] Sonntag E., Hamilton S.T., Bahsi H., Wagner S., Jonjic S., Rawlinson W.D., Marschall M., Milbradt J. (2016). Cytomegalovirus pUL50 is the multi-interacting determinant of the core nuclear egress complex (NEC) that recruits cellular accessory NEC components. J. Gen. Virol..

[27] Hellberg T., Passvogel L., Schulz K.S., Klupp B.G., Mettenleiter T.C. (2016). Nuclear Egress of Herpesviruses: The Prototypic Vesicular Nucleocytoplasmic Transport. Adv. Virus Res..

[28] Bigalke J.M., Heldwein E.E. (2017). Have NEC Coat, Will Travel: Structural Basis of Membrane Budding During Nuclear Egress in Herpesviruses. Adv. Virus Res..

[29] Lye M.F., Wilkie A.R., Filman D.J., Hogle J.M., Coen D.M. (2017). Getting to and through the inner nuclear membrane during herpesvirus nuclear egress. Curr. Opin. Cell Biol..

[30] Sharma M., Bender B.J., Kamil J.P., Lye M.F., Pesola J.M., Reim N.I., Hogle J.M., Coen D.M. (2015). Human cytomegalovirus UL97 phosphorylates the viral nuclear egress complex. J. Virol..

[31] Steingruber M., Keller L., Socher E., Ferre S., Hesse A.M., Coute Y., Hahn F., Buscher N., Plachter B., Sticht H. (2019). Cyclins B1, T1, and H differ in their molecular mode of interaction with cytomegalovirus protein kinase pUL97. J. Biol. Chem..

[32] Lemnitzer F., Raschbichler V., Kolodziejczak D., Israel L., Imhof A., Bailer S.M., Koszinowski U., Ruzsics Z. (2013). Mouse cytomegalovirus egress protein pM50 interacts with cellular endophilin-A2. Cell Microbiol..

[33] Marschall M., Marzi A., aus dem Siepen P., Jochmann R., Kalmer M., Auerochs S., Lischka P., Leis M., Stamminger T. (2005). Cellular p32 recruits cytomegalovirus kinase pUL97 to redistribute the nuclear lamina. J. Bioli. Chem..

[34] Milbradt J., Auerochs S., Sticht H., Marschall M. (2009). Cytomegaloviral proteins that associate with the nuclear lamina: Components of a postulated nuclear egress complex. J. Gen. Virol..

[35] Milbradt J., Kraut A., Hutterer C., Sonntag E., Schmeiser C., Ferro M., Wagner S., Lenac T., Claus C., Pinkert S. (2014). Proteomic analysis of the multimeric nuclear egress complex of human cytomegalovirus. Mol. Cell. Proteom..

[36] Jha B.K., Salunke D.M., Datta K. (2002). Disulfide bond formation through Cys186 facilitates functionally relevant dimerization of trimeric hyaluronan-binding protein 1 (HABP1)/p32/gC1qR. Eur. J. Biochem..

[37] Tischer B.K., Smith G.A., Osterrieder N. (2010). En passant mutagenesis: A two step markerless red recombination system. Methods Mol. Biol..

[38] Vu A., Poyzer C., Roller R. (2016). Extragenic Suppression of a Mutation in Herpes Simplex Virus 1 UL34 That Affects Lamina Disruption and Nuclear Egress. J. Virol..

[39] Funk C., Ott M., Raschbichler V., Nagel C.H., Binz A., Sodeik B., Bauerfeind R., Bailer S.M. (2015). The Herpes Simplex Virus Protein pUL31 Escorts Nucleocapsids to Sites of Nuclear Egress, a Process Coordinated by Its N-Terminal Domain. PLoS Pathog..

[40] Changotra H., Turk S.M., Artigues A., Thakur N., Gore M., Muggeridge M.I., Hutt-Fletcher L.M. (2016). Epstein-Barr virus glycoprotein gM can interact with the cellular protein p32 and knockdown of p32 impairs virus. Virology.

[41] Wang Y., Yang Y., Wu S., Pan S., Zhou C., Ma Y., Ru Y., Dong S., He B., Zhang C. (2014). p32 is a novel target for viral protein ICP34.5 of herpes simplex virus type 1 and facilitates viral nuclear egress. J. Biol. Chem..

