# Supplementary material for: Patterns of Autologous and Nonautologous Interactions between Core Nuclear Egress Complex (NEC) Proteins of α-, β- and γ-Herpesviruses
Source: Viruses. 2020 Mar 11;12(3):303. doi: 10.3390/v12030303 (PMC7150769; doi:10.3390/v12030303)
Supplement: Supplementary file 1 [file viruses-12-00303-s001.pdf]

viruses

MDPI

Supplementary Materials

Patterns of autologous and nonautologous interactions between core nuclear egress complex (NEC) proteins of α-, β- and γ-herpesviruses

Sigrun Häge [1], Eric Sonntag [1], Eva Maria Borst [2], Pierre Tannig [1], Lisa Seyler [3], Tobias Bäuerle [3], Susanne M. Bailer [4], Chung-Pei Lee [5], Regina Müller [1], Christina Wangen [1], Jens Milbradt [1] and Manfred Marschall [1,*]

[1] Institute for Clinical and Molecular Virology, Friedrich-Alexander University of Erlangen-Nürnberg (FAU), zip code Erlangen, Germany; sigrun.haege@fau.de (S.H.); ericsonntag@web.de (E.S.); pierre.tannig@uk-erlangen.de (P.T.); mueller.regina@uk-erlangen.de (R.M.); christina.wangen@uk-erlangen.de (C.W.); jens.milbradt@lgl.bayern.de (J.M.); manfred.marschall@fau.de (M.M.)

[2] Institute of Virology, Hannover Medical School, zip code Hannover, Germany; borst.eva@mh-hannover.de (E.M.B.)

[3] Institute of Radiology, University Medical Center Erlangen, zip code Erlangen, Germany; lisa.seyler@uk-erlangen.de (L.S.); tobias.baeuerle@uk-erlangen.de (T.B.)

[4] Fraunhofer Institute for Interfacial Engineering and Biotechnology, Stuttgart, Germany, and Institute for Interfacial Engineering and Plasma Technology IGVP, University of Stuttgart, zip code Stuttgart, Germany; susanne.bailer@igvp.uni-stuttgart.de (S.M.B.)

[5] School of Nursing, National Taipei University of Nursing and Health Sciences, Taipei zip code, Taiwan; chungpei@ntunhs.edu.tw (C.P.L.)

* Correspondence: manfred.marschall@fau.de; Tel.: +49-9131-8526089

Received: 14 February 2020; Accepted: 6 March 2020

Table S1. Oligonucleotide primers used in this study. The following information is given by the sequence description: tag sequences (capital letters, italic), translational start or stop codons (capital letters, underlined), restriction sites (capital letters, bold), additional bases (lower case letters) and coding sequences (capital letters).

Primer	Sequence (5′→ 3′)
5-M50-EcoRI	tag**GAATTC**ATGGAGATCGACAAGAATGTGG
3-M50-HA-XhoI	tag**CTCGAG**TCA*AGCGTAATCTGGAACATCGTATGGGTA*CGGATGACCCGCCGAACGG
5-M53-EcoRI	tag**GAATTC**ATGTTTAGGAGCCCGGAGGGAGAG
3-M53-Flag-XbaI	tag**TCTAGA**TCA*CTTGTCGTCATCGTCTTTGTAGTC*CAACGAGTAACTCTCGAAC
5-BFRF1-BamHI	tag**GGATCC**ATGGCGAGCCCGGAAGAGAGG
3-BFRF1-HA-XhoI	tag**CTCGAG**TCA*AGCGTAATCTGGAACATCGTATGGGTA*GGTCCACCTCAGAAACATCAGG
5-BFLF2-BamHI	tag**GGATCC**ATGGCCCCGGTCACCCCAG
3-BFLF2-Flag-XhoI	tag**CTCGAG**TCA*CTTGTCGTCATCGTCTTTGTAGTC*CTGTTTATTTTCCAAAATGAGCTGGG
5-ORF24-BamHI	tag**GGATCC**ATGTCACGGAGAACGTATGTACG
3-ORF24-HA-XhoI	tag**CTCGAG**TCA*AGCGTAATCTGGAACATCGTATGGGTA*TTTCCAGAAAAGCACCGCC
5-ORF27-BamHI	tag**GGATCC**ATGCATTTAAAGCCTACCAG
3-ORF27-Flag-XhoI	tag**CTCGAG**TCA*CTTGTCGTCATCGTCTTTGTAGTC*CCGAGGAGGAACAAAGTCATC
5′-DM50-insUL50	GGCGGCGGTCGGCGAGGGGGAGCGGCGGCGAGGCACCTGGCACGGCGACAGATGAGGG ACTCAGTCGCGGTGTGCGGAG
3'-DM50-insUL50	GGTCGGCTCGGGCGGCGCCACTCGGACGGCGGCGAGCTCATCCGCGGCGGCGCCGGCGC GATGGAGATGAACAAGGTTCTCC

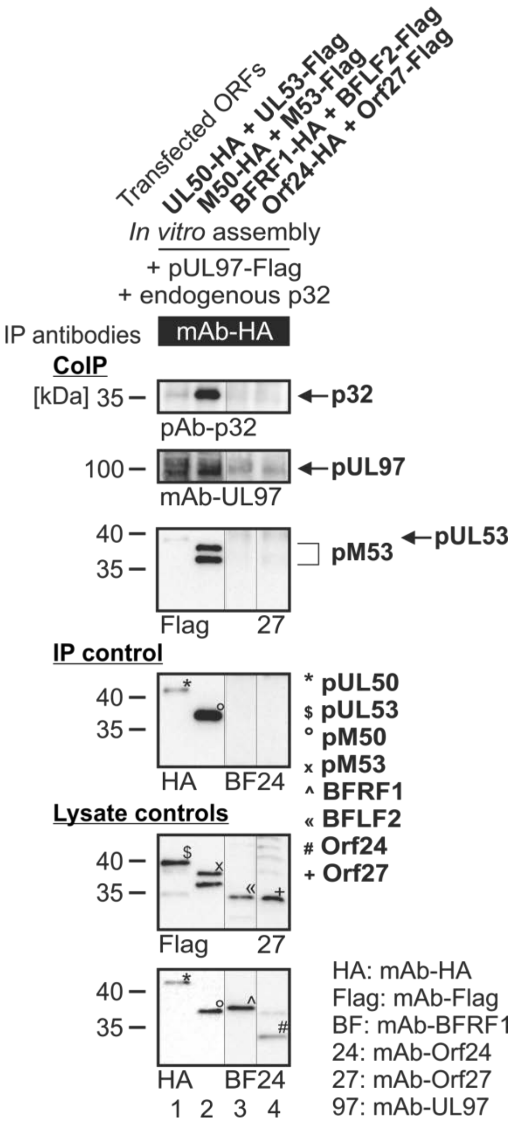

Figure S1. Assembly-based CoIP for core NECs with p32/gC1qR and pUL97. 293T cells were transiently transfected with HA-tagged pUL50, pM50, BFRF1, Orf24; Flag-tagged pUL53, pM53, BFLF2, Orf27 or pUL97. At three d p.t., cells were lysed and for assembly lysates were mixed and incubated at 4° overnight. HA- or Flag-tagged proteins were immunoprecipitated using mAb-HA and incubated with the lysate containing pUL97-Flag for 3 h. Lysate controls taken prior to the IP and CoIP samples were subjected to standard Wb analysis using tag- and protein-specific antibodies as indicated.

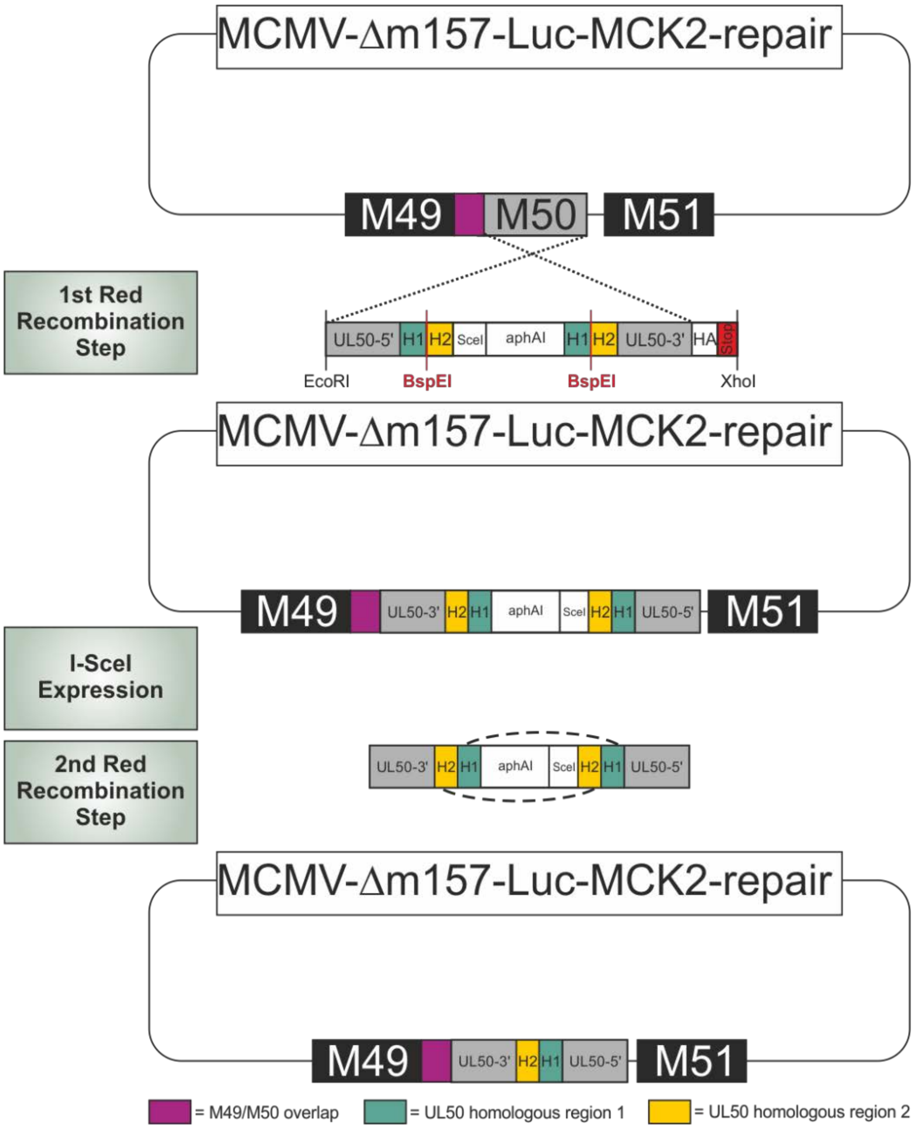

Figure S2. Schematic representation of the generation of recombinant MCMV. For the insertion of ORF UL50, a UTC harboring the positive selection marker (*aphAI* kanamycin cassette) and flanked by UL50 homologous regions was generated. In order to retain the ORF M49, the part shared by M49 and M50 (marked in purple) was not replaced. By PCR amplification, the homologous regions required for insertion of the UTC were added and transferred into recombination-active bacteria. After the first step of recombination, the ORF M50 was substituted by the UTC. After induction of I-*SceI* expression and the second step of recombination, the kanamycin cassette was completely removed from the BACmid.

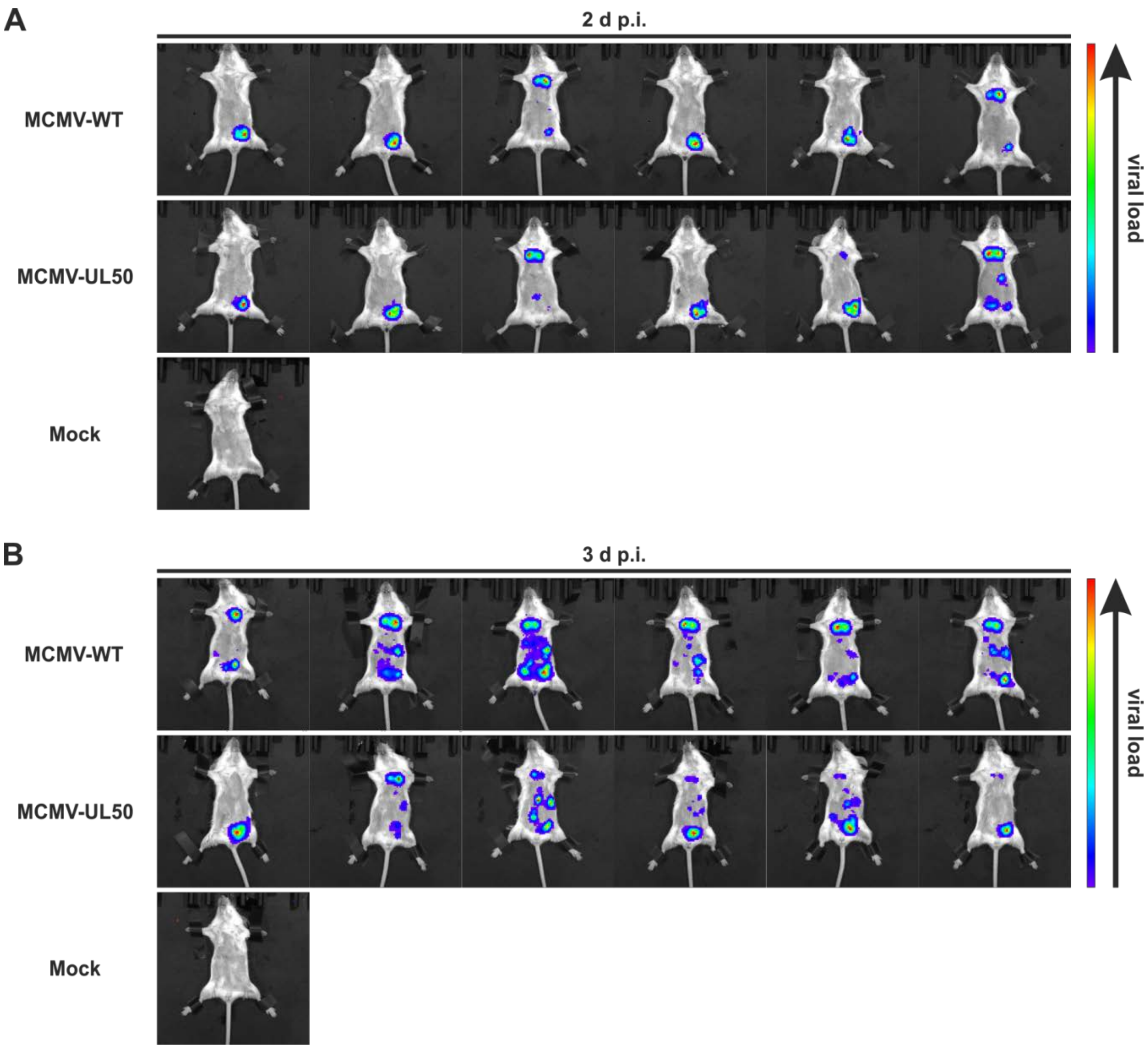

Figure S3. *In vivo* detection of virus replication. At (**A**) 2 d p.i. or (**B**) 3 d p.i. mice were anesthetized for analyzing virus replication and spread by *in vivo* imaging. 150 mg/kg body weight luciferin was injected i.p. and detection was performed 10 min after injection.
